# Divergence in Dialogue

**DOI:** 10.1371/journal.pone.0098598

**Published:** 2014-06-11

**Authors:** Patrick G. T. Healey, Matthew Purver, Christine Howes

**Affiliations:** Cognitive Science Research Group, School of Electronic Engineering and Computer Science, Queen Mary University of London, London, United Kingdom; University of Leicester, United Kingdom

## Abstract

One of the best known claims about human communication is that people's behaviour and language use converge during conversation. It has been proposed that these patterns can be explained by automatic, cross-person priming. A key test case is structural priming: does exposure to one syntactic structure, in production or comprehension, make reuse of that structure (by the same or another speaker) more likely? It has been claimed that syntactic repetition caused by structural priming is ubiquitous in conversation. However, previous work has not tested for general syntactic repetition effects in ordinary conversation independently of lexical repetition. Here we analyse patterns of syntactic repetition in two large corpora of unscripted everyday conversations. Our results show that when lexical repetition is taken into account there is no general tendency for people to repeat *their own* syntactic constructions. More importantly, people repeat *each other's* syntactic constructions *less* than would be expected by chance; i.e., people systematically diverge from one another in their use of syntactic constructions. We conclude that in ordinary conversation the structural priming effects described in the literature are overwhelmed by the need to actively engage with our conversational partners and respond productively to what they say.

## Introduction

It is widely reported that people's behaviour tends to converge during conversation; amongst other things body posture, movements, speech rhythm, speech rate, accent and facial expressions all tend to become more similar [1]–[3]. Early accounts of these phenomena emphasised the role of strategic social processes in promoting convergence [2]. However, more recent theories have proposed that convergence can be explained in terms of automatic, cross-person priming mechanisms [4]–[6]. The advantage of a priming account is that it promises a relatively simple, computationally inexpensive explanation of the basic mechanisms underpinning communication that is compatible with implementation by the human ‘mirror neuron’ system [6]–[9].

Recently, the reliability of some cross-person ‘social’ priming effects has been called into question [10], [11]. One source of concern is that, unlike conventional priming effects, social priming often appeals to activation of high level mental representations and involves effects that persist over relatively long time-scales and across different contexts e.g. reading words associated with being old as a prime for ‘older’ patterns of walking several minutes later.

Structural priming, which plays an important role in priming-based accounts of communication, is less vulnerable to these concerns. It involves low-level structural representations and priming over short intervals between instances of language comprehension and production in the same modality. Structural priming is also insulated to some extent from conscious or strategic social goals. People are not normally aware of the syntactic structures they use in conversation nor whether they are matching each other's syntax [12]. This makes syntax a good candidate for automatic, cross-person priming processes. Partly because of this it has been accorded a key role in helping to drive the alignment of higher level cognitive representations during communication including the co-ordination of semantic content and situation models [5], [6]. It thus constitutes an important test case for priming-based models of communication.

Most of the psycholinguistic evidence for structural priming is drawn from experimental studies of self-repetition in which people process sequences of written or spoken sentences in isolation (see [13], [14] for reviews). Fewer studies have directly investigated whether people tend to repeat each other's syntax in conversational contexts.

The empirical evidence for structural priming in conversation comes from corpus analyses of syntactic repetition and from experimental studies of task-oriented dialogue. Here we argue that the patterns of other-repetition reported in these studies do not generalise to ordinary conversation. We present a new analysis of two corpora of ordinary spoken dialogues which shows that when we take patterns of lexical repetition into account people do not repeat their own syntax more than would be expected by chance. Moreover, people systematically diverge from one-another in their use of syntactic structures. These results are incompatible with the predictions of automatic priming-based models of communication and therefore undermine the claim that priming is the basic mechanism underpinning successful human interaction.

We argue that priming, and the patterns of repetition it predicts, provides a conservative model of communication that is unable to address how we engage productively with our conversational partners. Although the coherence of conversation depends on repeating some of each other's words this is not a ‘blind’, automatic process. Rather, successful communication seems to depend on the ability to selectively repeat some of our conversational partner's words in different syntactic contexts in order to produce the contrasts, elaborations and corrections that move a conversation forward.

### Corpus Evidence for Structural Convergence in Dialogue

Relatively few corpus studies of repetition address the specific question of whether people show a general tendency to repeat each other's syntax in conversation. Some use small sample sizes that don't generalise well (e.g. [15], eight conversations; [16] three conversations) or focus their analysis on particular subsets of syntactic structures [13], [17], [18]. The data sets also sometimes include non-conversational elements such as written and spoken monologue [13], [16], [18], [19] or include different genres of spoken data such as lectures, speeches and interviews [13], [16], [18].

Three studies that focus directly on other-repetition in spoken dialogue have produced inconclusive results. Reitter et al [20] find other-repetition of all syntactic structures is above chance in a face-to-face route description task but below chance in telephone conversations involving the alternating discussion of predefined topics. In contrast to this, a study focussing on five target syntactic constructions using the same telephone corpus finds other-repetition is above chance for three out of five constructions [19]. Gries [13] finds patterns of other-repetition above chance for two constructions: the prepositional object-direct object (or PO-DO) alternation and verb-particle placement. However this corpus includes written and spoken monologue and context specific situations such as legal cross-examinations and broadcast interviews. Re-analysis using only the unstructured dialogues from this data set, i.e. formal and informal face-to-face conversations and telephone calls, finds no effect of other-repetition [21].

### Experimental Evidence for Structural Convergence in Dialogue

The first experimental study of cross-person repetition in dialogue is provided by Levelt and Kelter [22], however this investigates word repetition not structural repetition. The strongest experimental evidence for cross-person structural repetition comes from Branigan and colleagues [23], [24]. Subjects are presented with a pictorial scene with a verb printed below, e.g. ‘give’. The picture can be equally well described by, for example,“The girl giving the book to the boy” (a Prepositional Object or PO structure) or “The girl giving the boy the book” (a Double Object or DO structure). If an experimental confederate and a naïve subject alternate in producing descriptions of a sequence of such scenes, the choice of one structure by the confederate systematically increases the likelihood that the naïve subject will choose the same structure for the next item they describe. Importantly, this effect is independent of lexical repetition as it is present even when the target verb is not repeated between the prime picture and the subsequent target picture [23]. Cleland and Pickering [25] also manipulate noun phrase structures (pre-nominal vs relative clause) as primes instead of verb phrases but find a less consistent structural priming effect.

For practical reasons experimental studies are only able to test a relatively small number of syntactic constructions. The need for experimental control also inevitably limits the naturalness of the interaction. Confederates in these studies follow a script and the naïve participants are instructed that they can only describe an item or say “Please repeat” which considerably restricts the dialogue.

In summary, the strongest evidence in support of structural priming effects is based on task-oriented dialogues, gathered in controlled environments and for a limited number of syntactic structures. Consequently, the prediction that structural priming should lead to general convergence in ordinary conversation has not been directly tested. To address this we analyse patterns of syntactic repetition across all syntactic structures in two large corpora of unscripted, open-ended conversations gathered in a variety of everyday contexts.

### Correlations Between Syntactic and Lexical Repetition

The topical coherence of conversation depends on recurrent references to people, places, activities or events [15], [16] and these repetitions automatically increase the likelihood of syntactic repetition. For example, if a verb of a particular syntactic type (e.g. transitive or ditransitive) is repeated this also constrains the syntactic structure of the repetition. As a result, tests for independent effects of syntactic repetition need to correct for the correlation between word repetition and syntactic repetition [13], referred to in experimental studies as the ‘lexical boost’ effect [14], [26]. Existing studies do not directly correct for this correlation in their estimates of syntactic repetition effects [19], [20], [22], [27] although some studies mitigate it by excluding verbatim repetition of phrases [20]. To address this we include word repetition directly as a covariate in our analysis of syntactic repetition.

### Hypotheses

Priming-based models of communication predict that there should be a general tendency for different linguistic structures to repeat across turns in conversation. This is expected to occur at multiple levels of linguistic representation such as phonetics, phonology, words, syntax, semantics and situation models and priming at one level is expected to facilitate priming at other levels through a process referred to as percolation [5], [6]. This helps alignment at one level of representation promote alignment at another. Priming effects are expected to be strongest immediately after a representation has been activated but then decay as the distance from the prime, measured in time or intervening words or turns increases. This leads to three key predictions about structural priming:

1. Repetition: people should repeat their own and each other's syntactic structures more often than chance,
2. Percolation: priming at one level (e.g. syntax) should facilitate priming at another (e.g. words) and
3. Decay: the likelihood of repetition of a syntactic structure should decrease with distance from a prime.

## Methods

To test these predictions we analyse the levels of syntactic and lexical repetition over sequences of turns in spoken face-to-face conversation and compare these with the levels of repetition that would be expected by chance.

### Materials and Design

We use two published corpora: the Diachronic Corpus of Present-Day Spoken English [28] and the British National Corpus [29] – Table 1 shows a summary of the data used. Both corpora contain transcriptions of spontaneous conversations based on mobile tape recordings collected by people sampled from different age groups, locations and social classes in the UK. Two corpora are used to ensure sufficient statistical power and as a cross-check on the possible influence of different parse trees and different grammar formalisms on estimates of syntactic repetition. The DCPSE is hand-annotated with syntactic parse trees [30]. We produced machine parsed equivalents for the larger BNC corpus by parsing with a Combinatory Categorial Grammar (CCG) [31] using a computational parser [32]. Examples of the different parse trees the two approaches produce for the same utterances (shown in Table S1 in File S1) are provided in Figures S2 and S3 in File S1.

**Table 1:** Summary of Corpus Samples.

Corpus	Syntactic Annotation	Number of Turns	Number of People
DCPSE	Hand Coded Parse Trees	6616	92
BNC	Machine Coded CCG Trees	95169	2020

### Procedure

For each person in each conversation we calculate the similarity between each turn they produce and each of the preceding five turns by either their interlocutors (other-similarity) or themselves (self-similarity). This provides a moving window of syntactic and lexical similarity of ten conversational turns that is passed over the whole conversation (see Table 2). Turns that are unmatched because they occur near the start of a conversation are recorded in the data files as missing values.

**Table 2:** Example Real Conversation and Corresponding ‘Chance Other’ Sequence and Associated Other-Similarity Values.

Real Conversation (DCPSE: DI-B33-1)	Lexical Other Similarity	Syntactic Other-Similarity
	T-1	T-2	T-3	T-4	T-5	T-1	T-2	T-3	T-4	T-5
**A**: ed oh god she's still talking isn't she laura never gets off the phone										
**B**: she doesn't Laura's amazing once on the phone really I've never heard	0.13					0.21				
**A**: you know I had three people try to ring me and constantly engaged here apparently three people										
**B**: really Laura's amazing when she gets on that phone she just does not get off	0.00	0.12				0.12	0.20			
**A**: I know										
**B**: I've never heard anybody spend so much time on the phone and such useless drivel most of the time	0.10	0.03	0.08			0.20	0.19	0.26		
**A**: I feel very sorry for the person talking to her										
**B**: yeah really	0.00	0.00	0.00	0.00		0.17	0.00	0.16	0.17	
**A**: it looks a good vehicle yeah										
**B**: it does very handy	0.14	0.11	0.00	0.00	0.00	0.12	0.20	0.33	0.12	0.14
**Randomised 'Chance Other' Sequence**										
**A**: ed oh god she's still talking isn't she laura never gets off the phone										
***V**: no but at home what do they speak*	0.00					0.38				
**A**: you know I had three people try to ring me and constantly engaged here apparently three people										
***W**: did they look at Forster's work as a whole*	0.00	0.03				0.18	0.27			
**A**: I know										
***X**: oh yeah I've got a big bag of uh recyclable sort of some time*	0.09	0.01	0.04			0.08	0.07	0.11		
**A**: I feel very sorry for the person talking to her										
***Y**: mm*	0.00	0.00	0.00	0.00		0.00	0.00	0.00	0.00	
**A**: it looks a good vehicle yeah										
***Z**: oh we must try it it was so good grilled*	0.20	0.00	0.00	0.01	0.01	0.33	0.34	0.33	0.12	0.15

Syntactic similarity (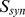) is calculated as the number of non-terminal syntactic structures (see Figure S1 in File S1) shared by pairs of turns (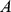 and 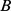) normalised for the total number of structures in both turns: 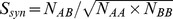. We include all complete subtrees that match across each pair of turns. Lexical similarity (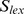) is calculated as the number of matching word pairs 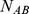 in each pair of turns 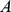 and 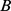 normalised for the total number of words in the two turns combined: 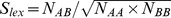. This yields similarity values for pairs of turns that vary between 0 for no match and 1 for a verbatim repeat. Examples of the similarity measures are provided in Table 2 and worked examples of the calculations are provided in the supplementary materials (see Figures S2 and S3 in File S1).

The software used to calculate similarities, perform corpus randomisation and produce machine-generated syntactic parse trees for the BNC is available under GPL license on: http://sourceforge.net/projects/diasim/. A copy of the CCG parser is available on: http://svn.ask.it.usyd.edu.au/trac/candc. The syntactic parse trees used for the DCPSE are part of the official DCPSE distribution.

To measure how much syntactic and lexical repetition occurs by chance we create randomised ‘Chance Other’ and ‘Chance Self’ conversations by randomly re-pairing turns. Chance Other consists of each person's real turns in sequence interleaved with turns randomly sampled from the rest of the corpus. As Table 2 illustrates, turns randomly combined in this way still show significant lexical and syntactic matches. Chance Self consists of each person's real turns in sequence paired with a random re-ordering of those turns subject to the constraint that no turn is matched with itself. This ensures that both the sample of people and language are counterbalanced across the real and chance ‘conversations’ so that the specific contribution of interaction to syntactic repetition can be separated out.

## Results

**Figure 1:**
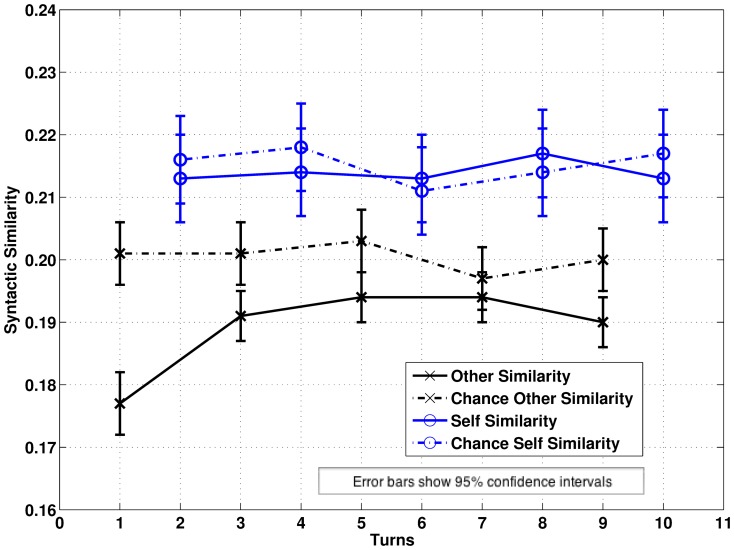
Estimated Marginal Means for Patterns of Syntactic Self-Similarity and Other-Similarity in a Ten Turn Window in the DCPSE.

**Figure 2:**
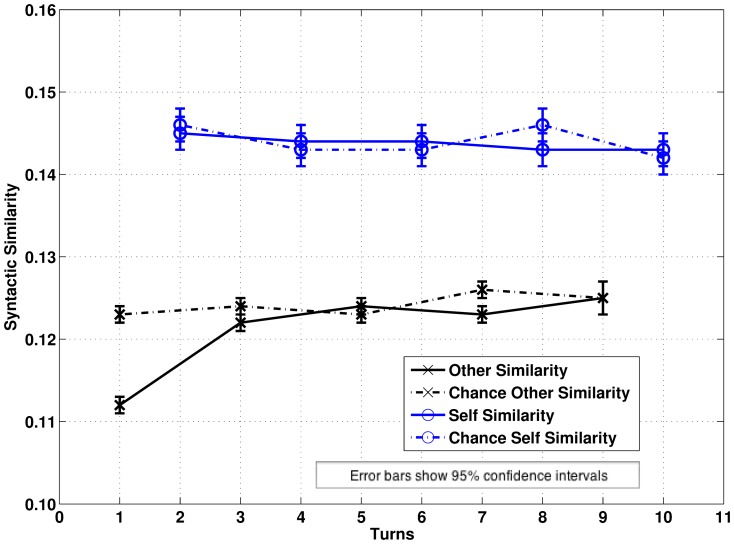
Estimated Marginal Means for Patterns of Syntactic Self-Similarity and Other-Similarity in a Ten Turn Window in the BNC.

Structural priming effects are tested in four General Linear Mixed Models (GLMM) of average cross-turn syntactic similarity for each person with Conversation (Real vs. Chance) and Distance (−1 to −5 turns from target) as repeated fixed factors and Subjects as a random intercept. Lexical Similarity (to Self or to Other respectively) is also included as a covariate to separate effects of syntactic similarity due to word repetition. A criterion level of p
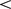
0.05 is adopted throughout though more precise p-values are reported for completeness. Bonferroni sequential adjustment is used throughout for multiple comparisons. The overall pattern of results is shown in Figure 1 (DCPSE) and Figure 2 (BNC). Note that the overall levels of syntactic match are lower for the BNC because of the greater variety of parse trees generated by the CCG parser.

In both corpora syntactic other-similarity is strongly conditioned by lexical similarity (DCPSE: 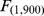 = 228.798, p
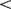
0.001, 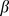 = +0.927; BNC: 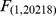 = 1860, p = 0.000, 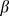 = +0.446). The DCPSE shows a main effect of Conversation (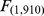 = 8.7, p
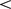
0.001) with syntactic other-similarity *lower* in real conversations compared to chance, but no other effects. As Figure 1 shows, syntactic repetition is below chance compared to the immediately preceding turn but tends to rise towards chance as distance increases (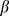 for pairwise comparisons at each level of distance: T-1 −0.024, p
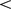
0.001; T-2 −0.010, p = 0.034; T-3 0.002, p = 0.040; T-4 0.003, p = 0.473; T-5 0.009, p = 0.041). The BNC shows no main effect of Conversation (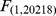 = 3.2, p = 0.073) but there is a Conversation 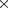 Distance Interaction (BNC: 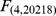 = 5.0, p = 0.001) and a Lexical Similarity 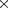 Distance Interaction (BNC: 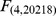 = 2.43, p = 0.045). As Figure 2 shows syntactic repetition is below chance in the next turn and then converges with chance (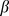 for pairwise comparisons: T-1 −0.011, p
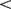
0.001; T-2 −0.002, p = 0.181; T-3 0.001, p = 0.555; T-4 −0.003, p = 0.123; T-5 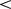
0.001, p = 0.862).

Syntactic self-similarity is also strongly influenced by lexical self-similarity (DCPSE: 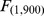 = 455, p
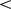
0.001, 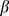 = +0.869; BNC: 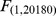 = 2609 p
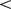
0.001, 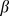 = +0.342). The DCPSE shows no main effect of Conversation but has a Conversation 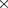 Distance Interaction (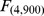 = 2.67, p = 0.031) and a Conversation 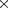 Distance 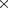 Lexical Self-similarity interaction (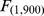 = 3.411, p = 0.009) reflecting a tendency for lexical self-similarity to boost syntactic self-similarity more in the real conversations at shorter distances. Focussed pairwise comparisons between real and chance self-similarity at each distance show no difference. The BNC also shows no main effect of Conversation but has a main effect of Distance (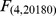 = 6.5, p
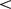
0.001) a Distance 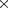 Lexical Self-similarity interaction (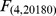 = 19.4, p
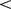
0.001) and a Conversation 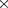 Distance 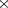 Lexical Self-similarity interaction (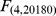 = 4.49, p = 0.001). This reflects an overall tendency for syntactic self-similarity boosted by lexical similarity to be higher at shorter distances in the real conversations. Focussed pairwise comparisons between real and chance self-similarity at each distance show no differences for either the DCPSE or the BNC.

## Discussion

These results confirm the correlation between word repetition and syntactic repetition and underline its strength. The biggest factor influencing syntactic repetition in this data is lexical repetition. As Table 2 illustrates, even randomly paired conversational turns show a degree of syntactic and lexical match. These results extend previous findings by demonstrating both repetition and a systemic correlation between syntax and word choice even in randomly paired utterances where interaction cannot have had any effect.

As argued above, the coherence of conversation depends on at least some lexical repetition. As a consequence it is necessary to take the correlation between words and syntax into account when attempting to estimate independent effects of syntactic repetition. In the present data lexical repetition is most common in the next turn and it is here that the effects on syntactic repetition are strongest.

When patterns of syntactic repetition are adjusted for the influence of lexical repetition they show a pattern that is incompatible with the predictions of priming-based models of communication. People do not repeat their own or each other's syntactic structures more than would be expected by chance. More importantly, people systematically *diverge* from their conversational partners in their use of syntax in the next turn. Although they sometimes respond using the same words they tend to use them in different syntactic contexts. This finding of local structural divergence is incompatible with the repetition prediction. It also runs counter to the percolation prediction since it shows lexical and syntactic other-repetition pull in opposite directions in adjacent turns. In addition it is incompatible with the prediction of decay since likelihood of other-repetition increases with distance although only rising towards chance.

Although this local pattern of divergence is opposite to the predictions of priming-based models it is compatible with observations about the functions of repetition identified in qualitative analyses of repetition in conversation. For example, Tannen [16] discusses repetition for functions such as humour, irony, expansion and elaboration. Schenkein [15] discusses the strategic use of repetition for performing sequences such as proposal, complaint, remedy. Repetition is also used to build contrastive formulations e.g. to turn a statement into a question, to introduce a disagreement, to appraise a proposal and to make corrections (e.g. [15], [16], [33], [34]). An example from the current analysis: A: “And it's Eileen's anniversary as well today.” B: “Oh bugger Eileen!” (DCPSE, KB1).

In these cases people repeat each other's words but in different syntactic contexts to produce the contrasts, elaborations and evaluations that sustain the forward momentum of conversation. This variety of uses of repetition is difficult to explain by reference to an automatic priming or ‘mirroring’ mechanism. Models which take the interaction of syntax and semantics with dialogue structure into account, and show how one type of contribution often licenses a different type as a follow-up (e.g. questions licensing fragment answers), might do more to explain these contrasts (e.g. [35], [36]).

In view of claims that conversation is “extremely repetitive” and that priming is “ubiquitous” [14] it is worth noting that even the absolute levels of lexical repetition observed across turns in the data presented here are low (e.g. 9% in real conversations which is only 3% above the chance levels of matching observed in the BNC). Ordinary conversation appears to involve relatively little word repetition and where it occurs it is a heterogeneous phenomenon. Although some words are repeated over 90% are not and a full account of successful conversation must be able to explain both.

The present results address only the general prediction that all syntactic structures should tend to repeat across turns in a conversation. This does not rule out the possibility that different syntactic structures follow different patterns. The prepositional object and double object constructions that are most strongly associated with structural priming are relatively rare in ordinary conversation and it is possible that this rarity itself may enhance the likelihood of repetition (see [37]). Nonetheless, our assumption is that the divergence effect observed here reflects the fact that the demands of constructive engagement with a conversational partner normally overwhelm the structural priming effects demonstrated in laboratory-based studies.

## Conclusions

Our results show that in ordinary dialogue people systematically diverge from one another in their use of syntactic structures in adjacent turns. This is incompatible with a structural priming account of syntactic co-ordination in dialogue and challenges the more general claim that automatic resource free priming provides the basic mechanism underpinning successful human communication.

## Supporting Information

File S1 **Similarity Calculations.** File S1 contains: **Figure S1**. Example syntax tree with subtrees. **Table S1**. Example DCPSE turn pairs: Real Conversation and Corresponding ‘Chance Other’ Sequence. **Figure S2**. Example DCPSE trees with matching subtrees and words highlighted. **Figure S3**. CCG trees (as used in the BNC) for the same sentences as Figure S2.
(PDF)
