# Supplementary material for: Divergence in Dialogue
Source: PLoS One. 2014 Jun 11;9(6):e98598. doi: 10.1371/journal.pone.0098598 (PMC4053332; doi:10.1371/journal.pone.0098598)
Supplement: File S1 — Similarity Calculations. File S1 contains: Figure S1. Example syntax tree with subtrees. Table S1. Example DCPSE turn pairs: Real Conversation and Corresponding ‘Chance Other’ Sequence. Figure S2. Example DCPSE trees with matching subtrees and words highlighted. Figure S3. CCG trees (as used in the BNC) for the same sentences as Figure S2. (PDF) [file pone.0098598.s001.pdf]

Similarity Calculations

To measure syntactic similarity between turns, we use the tree kernel calculation of [1]. For any tree, this metric is based on the subtrees within it corresponding to individual syntactic production rules: for example, the tree in Figure S1 below is composed of three subtrees corresponding to the production rules S → NP VP, NP → PN, VP → V NP and NP → DET CN. We omit the terminal (lexical) rules linking words to their syntactic categories PN → max, V → eats etc.

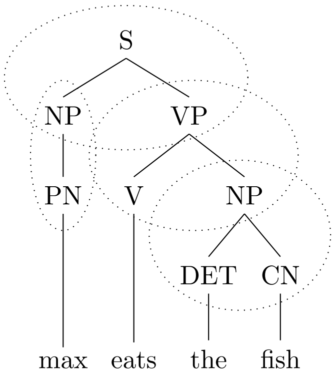

Figure S1: Example syntax tree with subtrees

For any two syntactic trees A and B, we define N_{AB} as the number of matching subtrees (subtrees with identical syntactic production rules) between A and B. The similarity between A and B is now defined as $N_{AB}/\sqrt{N_{AA} \times N_{BB}}$, where $\sqrt{N_{AA} \times N_{BB}}$ is a normalisation factor to account for the number of opportunities for matching within A and B. (N_{AA} is the number of matches when tree A is matched against itself – this will be at least equal to the number of subtrees in A, and will increase as subtrees are repeated identically within A). Lexical similarity uses the same calculation, with the exception that N_{AB} is now defined as the number of matching *words* between two *strings* A and B.

As the syntactic measure depends to some extent on the syntactic formalism and grammar assumed, we use two corpora with different approaches. First, the Diachronic Corpus of Present-Day Spoken English (DCPSE) [2] pairs sentences with hand-annotated parse trees using a phrase structure grammar [4]. Second, we parsed the British National Corpus (BNC) [3] using a state-of-the-art computational parser [5] based on Combinatory Categorial Grammar (CCG) [6]. We can thus compare results on two datasets using two formalisms, two grammars, and both manual and automatic parsing.

Our comparison in this paper is based on speaker turns: sequences of speech by one participant before another participant takes over. Both corpora also give a lower-level segmentation into sentences, where one turn may be composed of many sentences. Standard syntactic grammars apply at the sentence level, and the DCSPE pairs each sentence with its own parse tree; for the BNC, we also

parse each sentence separately. To calculate a similarity figure for a pair of turns, we then take the arithmetic mean of the pairwise similarities between the constituent sentences.

Figures S2 and S3 show examples of the trees, subtrees and similarity calculations in both syntactic formalisms. The example sentences are taken from the DCPSE and are as shown in Table S1:

Real Conversation	
A:	It looks a good vehicle yeah
B:	It does. Very handy

Randomised 'Chance Other' Sequence	
A:	It looks a good vehicle yeah
C:	*Oh we must try it. It was so good. Grilled*

Table S1: Example DCPSE turn pairs: Real Conversation and Corresponding 'Chance Other' Sequence

References

[1] Moschitti, A. Making tree kernels practical for natural language learning. In *Proceedings of the 11th Conference of the European Chapter of the Association for Computational Linguistics* (2006).

[2] Davies, M. The British component of the International Corpus of English (ICE-GB), release 2, and: Diachronic Corpus of Present-Day Spoken English (DCPSE), and: The International Corpus of English Corpus Utility Program (ICECUP), version 3.1 (review). *Language* **85**, 443–445 (2009).

[3] Quirk, R., Greenbaum, S., Leech, G. & Svartvik, J. *A Comprehensive Grammar of the English Language* (Longman, London, 1985).

[4] BNC. *The British National Corpus, version 3 (BNC XML Edition)* (Distributed by Oxford University Computing Services on behalf of the BNC Consortium., 2007). URL `http://www.natcorp.ox.ac.uk/`.

[5] Clark, S. & Curran, J. Wide-coverage efficient statistical parsing with CCG and log-linear models. *Computational Linguistics* **33**, 493–552 (2007).

[6] Steedman, M. *The Syntactic Process* (MIT Press, Cambridge, MA, 2000).

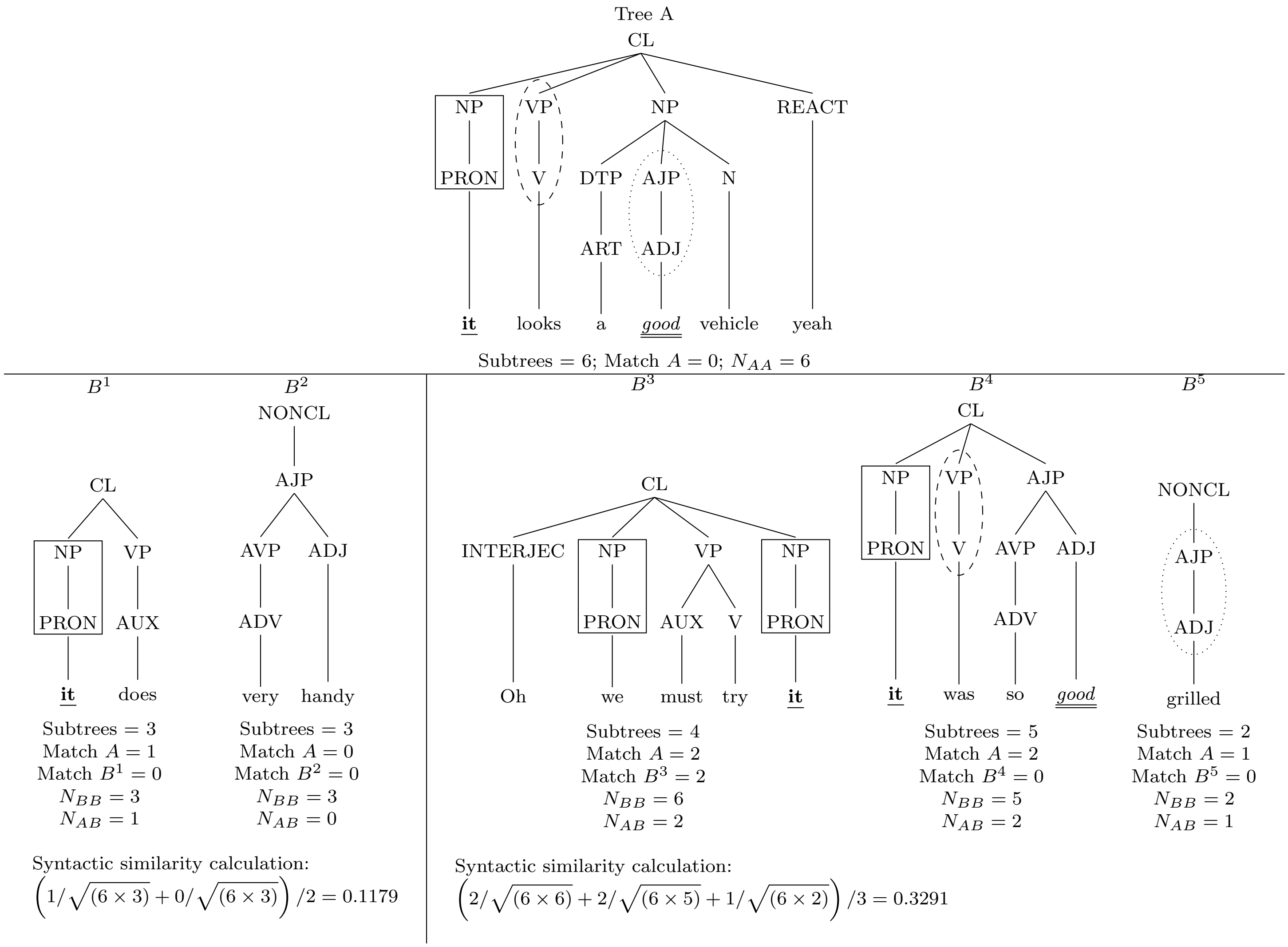

Figure S2: Example DCPSE trees with matching subtrees and words highlighted

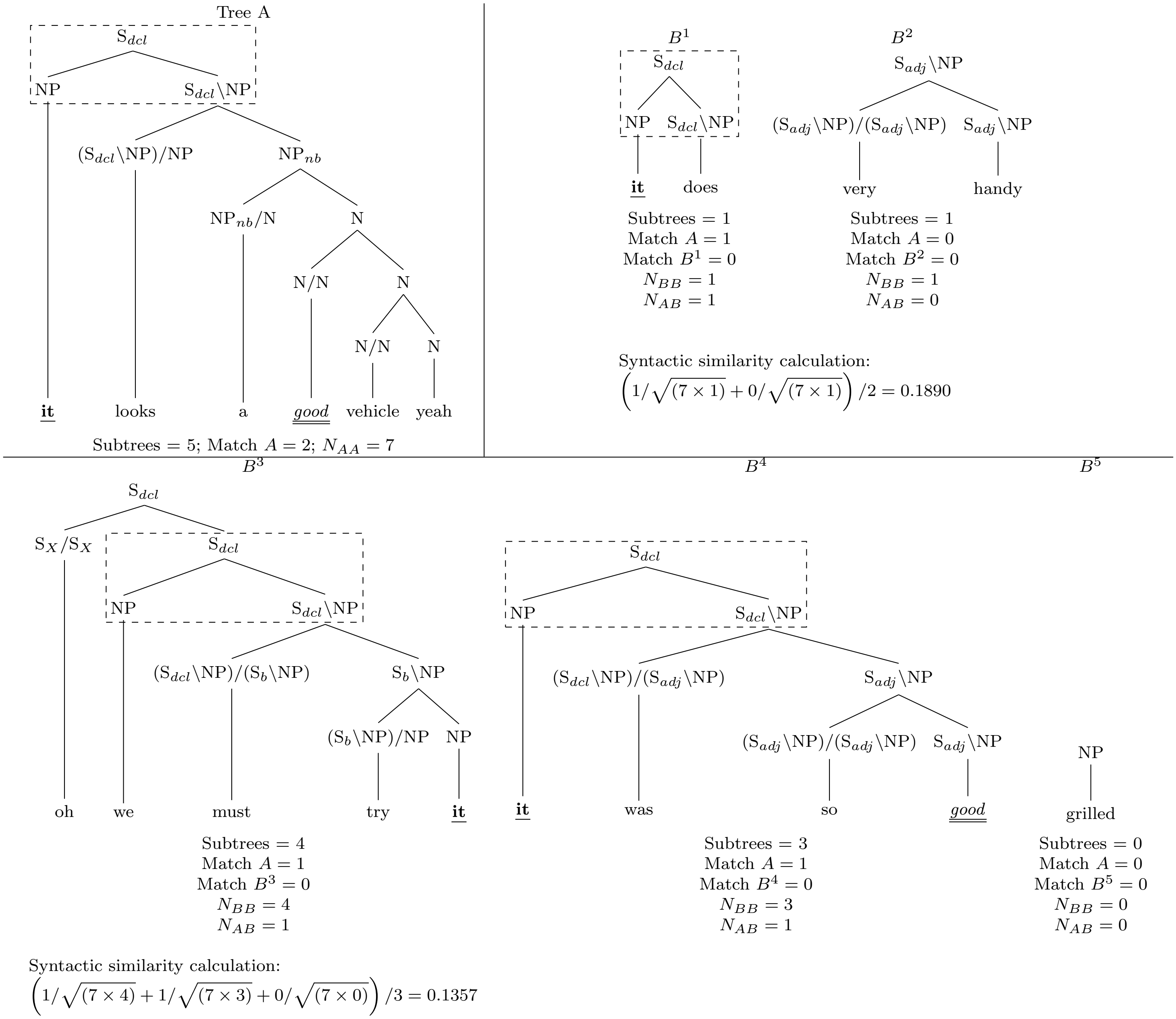

Figure S3: CCG trees (as used in the BNC) for the same sentences as Figure S2
